# Intrinsic motivation and false feedback reduce the low prevalence effect

**DOI:** 10.1186/s41235-025-00681-y

**Published:** 2025-10-09

**Authors:** Melina A. Kunar, Olugbemi Moronfolu, Rupam Jagota

**Affiliations:** Department of Psychology, The University of Warwick, Coventry, CV4 7AL UK

**Keywords:** Low prevalence, Motivation, Mammograms, Visual search

## Abstract

Previous research has found that people miss a large proportion of targets that appear rarely. This Low Prevalence (LP) Effect has implications for applied tasks such as mammography. The current study investigated whether the LP effect can be reduced by feedback and internal incentives, which affect motivation. Experiment 1 found that miss errors were reduced when participants were given false feedback about missed cancers; however, this also led to increased false alarm rates. Experiment 2 found no reduction in miss errors and an increase in false alarms when participants were given false feedback about both miss error and false alarm rates. Experiments 3 and 4 investigated the effect of signing a pledge on LP search. In Experiment 3, participants searched through a meaningless letter visual search task, whereas in Experiment 4 participants searched for a cancer in a mammogram. The results found that signing a pledge reduced the LP effect in the letter search task but not in the mammogram task. Experiment 5 found that the LP effect was reduced in the mammogram search task when the medical importance was emphasised to participants. Overall, the results demonstrate the importance of motivational factors in LP search.

## Significance statement

Infrequently appearing items, like cancers in mammograms, are often not detected due to their low prevalence. Our results showed that rare targets can be found more often if people are motivated by different factors. Giving people false feedback about the proportion of cancers they missed was shown to affect task performance, so that miss errors were reduced if participants were told they were performing badly. Signing a pledge motivated participants to better find the target, reducing miss errors in a non-medical search task, but had no effect when people searched for a cancer in a mammogram. However, emphasising the medical importance of the mammogram search task led to a reduction in the proportion of cancers missed. The results highlight the important role of motivation in low prevalence tasks, such as mammography.

## Introduction

People often perform visual search tasks in everyday life. For example, a person might search a room for their mobile phone or search for a friend in a coffee shop. Some visual search tasks have crucial health or safety aspects, such as search for a weapon during airport baggage screening or medical professionals searching a mammogram for a cancer. These latter tasks have the added complication that the target typically appears rarely—a factor that leads to poor search performance.

Wolfe et al. (2005) first examined the effects of target prevalence on search. They found that reducing the prevalence of the target from 50 to 1% led to a dramatic increase in miss errors from 7 to 30%, respectively. These high miss errors have consequences in applied search tasks where it is important to find the target (e.g. cancer in a mammogram; weapon in baggage screening). The ‘Low Prevalence’ (LP) effect has been shown to occur across a range of searches, including search for efficient features (e.g. a horizontal line among vertical lines, Rich et al., 2008), inefficient letter visual search tasks (e.g. Kunar et al., 2010; Rich et al., 2008; Russell & Kunar, 2012; Schwark et al., 2012), driving hazards (Kosovicheva, et al., 2023; Song & Wolfe, 2024), dynamic stimuli (Becker et al., 2024), baggage screening images (Wolfe et al., 2007) and medical images such as mammograms or skin lesions (Kunar et al., 2017, 2021, 2024; Kunar, 2022; Patterson & Kunar, 2024; Wolfe, 2022). Furthermore, when searching through medical images, the LP effect has been observed in experimental settings using observers with no medical training (e.g. Kunar et al., 2017, 2021, 2024; Wolfe, 2022), in experimental settings using observers with medical training (Wolfe, 2022), and in clinical settings with medical professionals (Evans et al., 2013).

Wolfe and Van Wert (2010) proposed a multiple decision model (MDM) to explain the LP effect. This model proposed that there are two factors affecting LP search. First, at low prevalence, the quitting threshold (at which a participant reaches a decision that a target is absent) is lower than when the target has a higher prevalence. This means that participants spend less time searching the display at LP compared with when the target has a high prevalence (HP). Second, at LP there is a shift in response criterion, so participants are less willing to respond that a target is present (i.e. showing a more conservative response bias). Evidence to support this has been found using Signal Detection Theory (SDT, Green & Swets, 1967; Macmillan & Kaplan, 1985), where, although sensitivity at LP does not change (as measured by *d’*), there is a large shift in response criterion (as measured by, *c,* e.g. Wolfe et al., 2007; Kunar et al., 2021, Russell & Kunar, 2012).

Given that a high miss error rate is far from ideal in tasks like baggage screening and medical imaging, many studies have investigated ways to reduce the LP effect. The results have been mixed. For example, separating the displays into two sections did little to eliminate the effect (Kunar et al., 2010). Neither did manipulations deliberately slowing down participants responses (Rich et al., 2008; Wolfe et al., 2007). However, some methods have been useful. For example, a double reading procedure where two observers searched the same display reduced the LP effect (Kunar et al., 2021; Wolfe et al., 2007) as did switching the task from a search task to a similarity matching task (Taylor et al., 2022). Furthermore, the LP effect was reduced when older adults were used as participants, due to a higher termination threshold before quitting search (Goodhew & Edwards, 2024) and has been found to be predicted by individual differences (Peltier & Becker, 2017, 2020).

Other avenues of research have investigated the role of reward, and feedback in LP search. First examining the effects of reward, previous research has shown that associating a target with an external reward, such as money, can affect visual search when the target has a high prevalence (e.g. Anderson & Yantis, 2012; Anderson et al., 2011; Laurent et al., 2015; Theeuwes et al., 2012), with value being identified as one of the factors affecting guidance in the influential Guided Search 6.0 model (Wolfe, 2021). Furthermore, it has been shown that financial rewards can affect performance in LP search. Navalpakkam et al. (2009) investigated the effect of monetary reward on target detection in an LP search task where participants were asked to find a target based on its line orientation. They found that when people were rewarded for their correct responses, and penalised for missing a target, the LP detriment was reduced. More recently, Hadjipanayi et al. (2023) found that when participants searched for multiple targets of different prevalence rates—with high financial reward to low prevalence targets—the LP effect was reduced.

The above research examined monetary incentives in LP search. In the current study, we examined whether it possible that rewards more intrinsic in nature also affect LP search (see Hollands & Marteau, 2013; Clark & Gilchrist, 2018; Franco et al., 2002, for examples of intrinsic motivation affecting human behaviour). We do this in two parts. In the first part, we examined whether feedback—in particular false motivational feedback telling people how well or poorly they were performing—affected LP search. In the second part, we examined whether making a pledge or receiving extra motivational information, affected LP search.

Let us start with the first part, which examines the effects of false feedback on LP search. Wolfe et al. (2007) found that providing correct feedback on a set of HP trials, embedded within an LP block, led to a decreased LP effect. Wolfe (2022) further found an effect of feedback when participants were asked to search medical images for skin lesions. The results found that giving participants feedback on a prior block of trials affected miss error rates on the subsequent block with an associated change in response bias. Of relevance to the work presented here, Schwark et al. (2012) manipulated false feedback in a letter visual search task. The feedback was mostly accurate; however, incorrect feedback was given on 20% of target-absent trials so that participants believed they missed more targets than they had. The results found that false feedback led to a change in response criteria, resulting in fewer overall miss errors and a larger proportion of false alarms compared to when no false feedback was given. However, please note that in Schwark et al. (2012)’s study the effect of feedback was coupled with a rewards scheme where participants were awarded points for correct responses and punished for incorrect ones. Thus, the effect of false feedback, in the absence of reward, is yet unknown. Furthermore, Schwark et al. (2012) only investigated false feedback given about miss errors. This naturally led to a change in miss errors, as people were motivated to reduce them, however, as participants were not given feedback about false alarms there was no similar incentive for these to be minimised. It could be that giving people false feedback about *both miss errors and false alarms* would lead to improved performance in both measures. We examined this here. Experiment 1 examined the effect of false feedback of *miss errors* with mammogram stimuli,^1^ in the absence of other reward incentives. Experiment 2 extended this work to investigate whether giving people false feedback for *both miss errors and false alarms* would lead to a reduction in both error types.

Now let us turn to the second part of our study. Here we examined the effects of other motivational factors on LP search, such as signing a pledge and giving extra motivational information. In terms of signing a pledge, previous research has found the act of making a pledge and/or signing your name changes people’s behaviour. Koessler (2022) found that having participants make a pledge, led to behaviour that enhanced commitment in a social public good game. Furthermore, signing one’s name has been shown to prime participants’ self-identity, thereby facilitating a person’s engagement with a task (Kettle & Häubl, 2011). It may also reduce cheating and dishonesty—although evidence for this is mixed (Kristal et al., 2020; Mazar et al., 2008; Peer et al., 2024).

Experiments 3 and 4 here investigated the effect of signing a pledge on LP search using different types of search tasks: a letter visual search and a mammogram search, as each type of search task may naturally prime different motivational factors. The former search task is inherently devoid of social good (nobody dies if you fail to find a letter T among Ls). However, finding a cancer in a mammogram is likely to contain intrinsic motivational factors due to the nature of the task. For example, in a medical setting, readers would be motivated to find a cancer given the negative health consequences of missing one. This is supported by recent research which has shown that vigilance decrements, well documented in the lab, may not occur in a clinical mammogram task, where detecting indicators of disease in a mammogram is intrinsically motivating (Taylor-Phillips et al., 2024). Furthermore, presenting medical images has been shown to affect motivation and cognitive factors leading to behavioural change (e.g. Hollands & Marteau, 2013).

Experiment 3 had participants sign a pledge declaring they will perform the task to the best of their ability before searching ‘meaningless’ letter visual search stimuli. If found to be successful, this could help reduce LP effects in tasks that may lack inherent motivation (e.g. checking for rotten fruit on a conveyor belt). In contrast, Experiment 4 had participants sign a pledge before searching more ‘meaningful’ mammogram search stimuli. To preview the results, we found that there was a benefit of participants signing a pledge, when searching through letter stimuli but not with the mammogram stimuli.

Lastly, Experiment 5 was used to investigate whether adding additional motivation to the mammogram search task led to better target detection. Here participants were given prior information explicitly emphasising the medical importance of the mammogram search task. In this case, the results showed that participants missed fewer cancers, compared to when they were not given this extra motivational information.

## Experiment 1

### Method

#### Transparency and openness

In all experiments, participants were recruited from the University of Warwick participant pool, had no prior training in reading mammograms and were either paid for their time or took part in the experiment for course credit. All participants gave informed consent before participation. Ethical approval for all studies was granted by the Humanities and Social Sciences Research Ethics Committee and the Department of Psychology’s Research Ethics Committee at the University of Warwick. In each of the experiments, 20 participants were recruited per condition, based on sample sizes from previous research (e.g. Kunar et al., 2017; Schwark et al., 2012; Wolfe et al., 2005, 2007). The data are available on the Open Science Framework (https://osf.io/2qwka/). All data were compiled in Microsoft® Excel® for Microsoft 365 MSO (Version 2112 Build 16.0.14729.20254) and imported into JASP (Version 0.13.1; JASP Team, 2021) for statistical analysis. The study design, hypotheses and analytic plan were not pre-registered. All manipulations, data exclusions and measures are reported.

#### Participants

Sixty participants took part in the experiment, with twenty participants per condition. Participants did not take part in any of the other experiments.

#### Stimuli and procedure

**Fig. 1:**
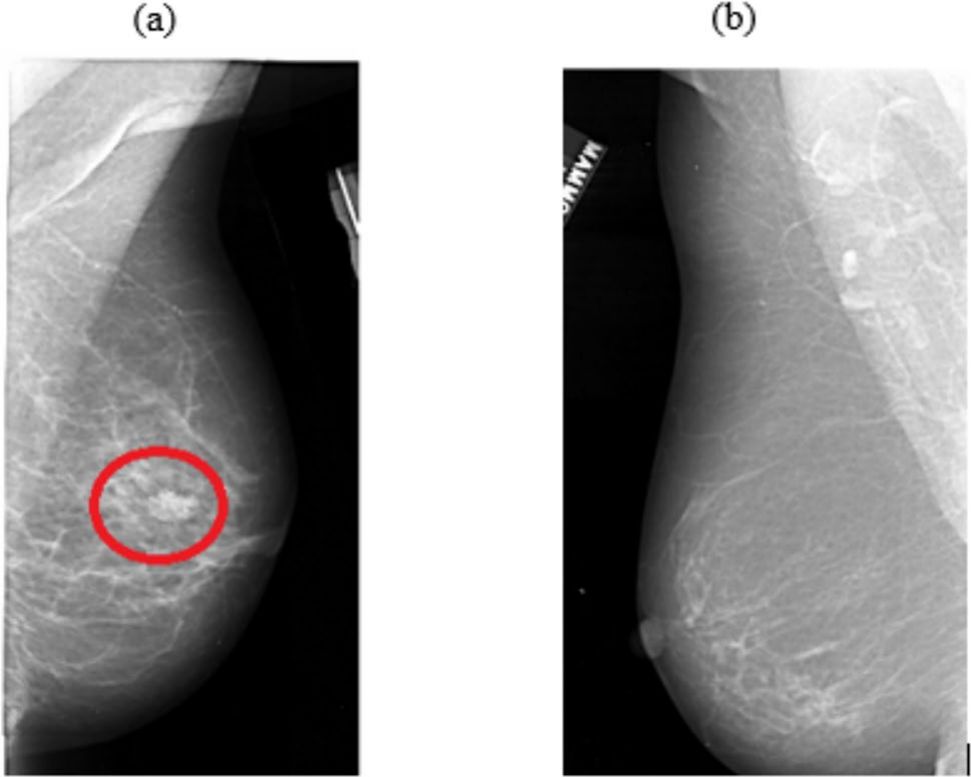
Examples of mammogram images **a** with cancer present and location circled **b** and without cancer. NB: the red circle around the cancer is for demonstration purposes and participants did not see this in the experiment

The experiment was programmed using BlitzMax and presented on a PC. The stimuli were comprised of 500 mammogram images and taken from the Digital Database for Screening Mammography (DDSM) database (Heath et al., 1998, 2001). All images were taken from the ‘normal’ mammogram category (those not containing a cancer). Images were presented in the centre of the display and subtended approximately 11 degrees by 19 degrees at a viewing distance of 57 cm (although the individual size of each image varied because they were real mammograms). Of all mammograms, 480 remained target-absent, and 20 had cancerous masses from the DDSM superimposed onto them. This created a 4% prevalence rate overall. For target present trials, four cancers masses were selected at random from cancer cases on the DDSM. These masses were then transposed onto mammograms that previously contained no cancer using imaging editing software so that each image contained one mass. The mass could appear on any area of the breast tissue, chosen at random (mimicking conditions in a clinical setting), provided that it was clearly distinguishable once fixated (see also Kunar et al., 2017, 2021, 2024; Kunar, 2022; Kunar & Watson, 2023; Patterson & Kunar, 2024). We used four masses so that participants were asked to search for a range of different perceptual stimuli, given that in radiology a mass can take on multiple different forms. However, as participants in these experiments did not have the same extensive medical training as radiologists, we needed to make sure the masses could be easily identifiable by non-medical readers. This follows the methodology of previous research (e.g. Kunar et al., Drew et al., 2012, 2020; Kunar, 2022; Patterson & Kunar, 2024). All mammogram images were created prior to the experiment. Example stimuli can be seen in Fig. 1.

In each trial, participants were shown a blank screen for 500 ms, at which point one of the mammogram images were shown and participants were asked to respond to the presence or absence of a cancer, by pressing the ‘m’ or ‘z’ key, respectively. The presentation order of the mammograms was randomised across participants.

In line with Fleck and Mitroff (2007) and to correct for motor errors, participants had the option of correcting their response to a trial during the succeeding trial. If a participant recognized that they had made an error, they were able to correct it on the following trial, by pressing the ‘Escape’ key. They would then proceed with the current trial as normal. Prior to the experiment, participants were given a training session where they were shown examples of the cancers both in isolation and on a mammogram, which they were asked to point out to the experimenter. Once they completed this training phase and the experimenter was confident that they could identify a cancer, participants completed a short practice session, before completing the experimental phase. If the participant still struggled with identifying the cancers after this, the briefing and practice process was repeated until the participant was successful in identifying the targets.

Participants took part in one of three conditions: a Control condition with no feedback, a condition where they were given false feedback that their performance was better than average (the Good condition) and a condition where they were given false feedback that their performance was worse than average (the Bad condition). For the False Feedback conditions, participants were informed of their performance after every block of trials, so that they saw a feedback statement four times throughout the experiment. The feedback statement read: “Most people miss 20% of cancers. You are missing *x*% of cancers.” (where *x* varied across feedback messages). The percentages used after each block were the same for each participant but varied across blocks. In the Good feedback condition, participants were told that their cancer miss-rate averaged at 16.5% (18%, 16%, 17% and 15% respectfully, after Blocks 1 to 4). In the Bad feedback condition, participants were told that their cancer miss-rate averaged at 40% (42%, 39%, 41% and 38% respectfully after Blocks 1–4).

In addition to cancer detection, this experiment also investigated if the feedback affected participants’ self-reported motivation. At the end of the experiment, participants rated three statements on a Likert scale. The statements were: (i) I *put a lot of effort into accurately detecting the presence of a cancer*; (ii) I *made sure that I scanned the mammogram carefully before responding* and (iii) *I felt motivated to do the experiment to the best of my ability*. Participants were asked to judge their agreement with these statements on a 5-point Likert scale (1, strongly disagree, to 5, strongly agree).

Trials that had Reaction Times (RTs) over 10,000 ms and those less than 200 ms were considered outliers and removed from data analysis. For all t-tests, Bayes factors analyses were reported (calculated with a Cauchy prior width of 0.707 using JASP version 0.13.1). The inclusion of Bayesian analyses gave the advantage of being able to evaluate evidence in support of the null hypothesis (Wagenmakers et al., 2018a). The recommendations of Jeffreys (1961) were adopted, in which a BF_10_ (which compares evidence of the alternative hypothesis to evidence for the null hypothesis) of 1 to 3 provides *anecdotal* evidence for the alternative, a BF_10_ of 3 to 10 provides *substantial* evidence for the alternative, a BF_10_ of 10 to 30 provides *strong* evidence for the alternative, a BF_10_ of 30 to 100 provides *very strong* evidence for the alternative and a BF_10_ of greater than 100 provides *decisive* evidence for the alternative. The inverse of these numbers (BF_01_) provides evidence in support the null hypothesis (Jarosz & Wiley, 2014). Please note that we only report Bayes statistics for the planned t-tests rather than for ANOVAs. Although Bayes statistics for t-tests are well established, best practices for ANOVAs are still under active debate with some unresolved discrepancies between Bayesian and frequentist results (e.g. Van Doorn et al., 2021; Wagenmakers, et al., 2018b).

### Results

**Fig. 2:**
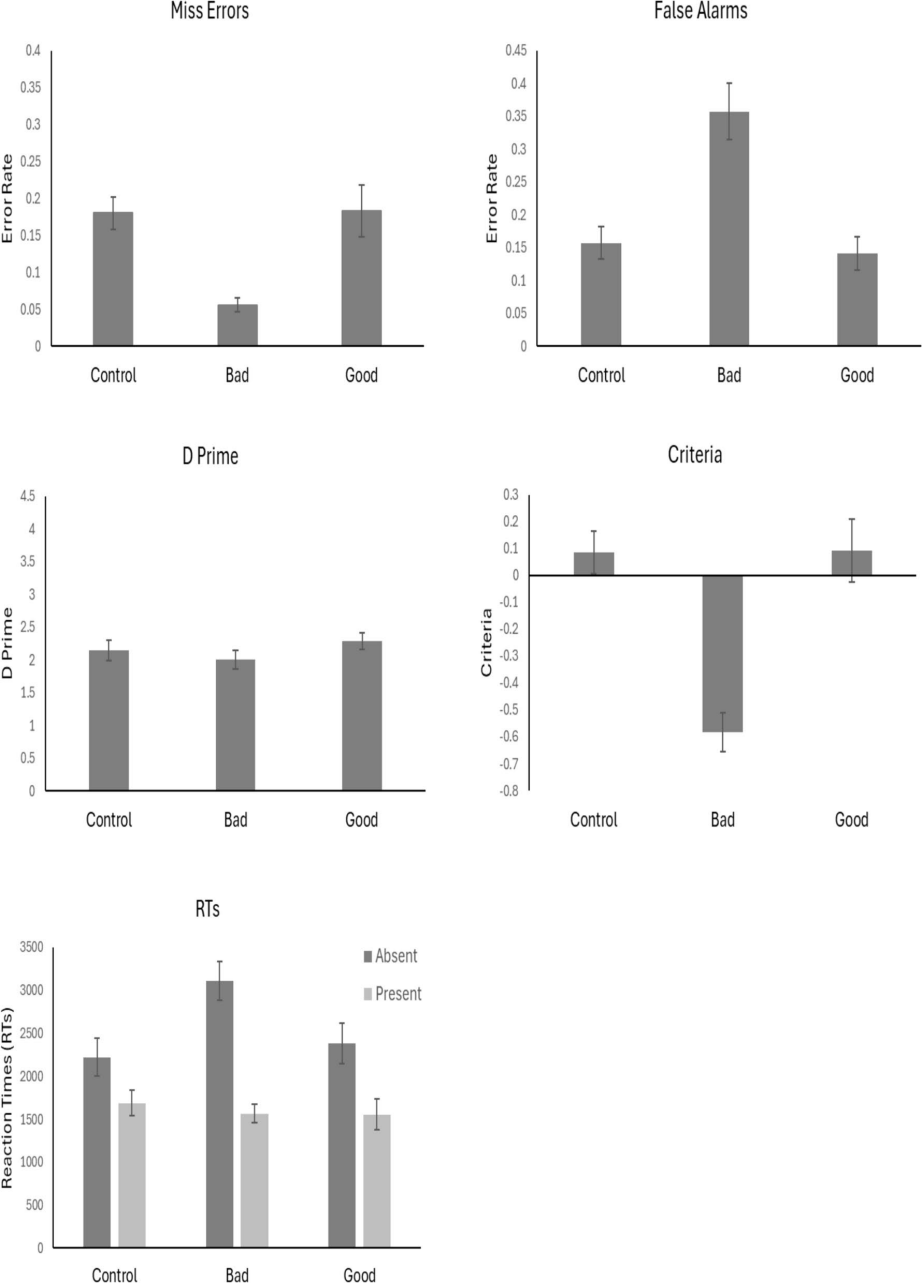
Mean values across conditions. *Note.* Error bars represent the standard error

The outlier procedure removed 2.5% of all data. Miss errors and false alarms were calculated by the proportion of cancers detected at the ‘final’ response (after any motor errors were removed). Given that miss errors and false alarms denote two different types of errors in applied tasks (e.g. mammography), we analysed these measures separately. Mean error rates, *d’, c* values and RTs for all conditions are presented in Fig. 2.

#### Miss errors

A one-way ANOVA showed a significant difference in miss errors across conditions, *F*(2, 57) = 8.74, *p* < 0.01, *η*^2^_*p*_ = 0.24. Planned t-tests showed that there were fewer miss errors in the Bad condition in comparison with the Control condition, *t*(38) = 5.14, *p* < 0.01, *d* = 1.63, with decisive evidence in support of the alternative, BF_10_ = 1839.42. The results did not show a difference in miss errors between the Control and the Good condition, *t*(38) = 0.07, *p* = 0.94, *d* = 0.02, with substantial evidence in support of the null, BF_10_ = 1/3.23. There were fewer miss errors in the Bad condition compared to the Good condition, *t*(38) = 3.50 *p* < 0.01, *d* = 1.11, with strong evidence in support of the alternative, BF_10_ = 27.00.

#### False alarms

A one-way ANOVA showed a significant difference in false alarms across conditions, *F*(2, 57) = 14.12, *p* < 0.01, *η*^2^_*p*_ = 0.33. Planned t-tests showed that there were a greater proportion of false alarms in the Bad condition in comparison with the Control condition, *t*(38) = 4.07, *p* < 0.01, *d* = 1.29, with decisive evidence in support of the alternative, BF_10_ = 106.30. The results did not show a difference in false alarms between the Control and the Good condition, *t*(38) = 0.43, *p* = 0.66, *d* = 0.14, with substantial evidence in support of the null, BF_10_ = 1/3.00. There were a greater proportion of false alarms in the Bad condition in comparison to the Good condition, *t*(38) = 4.35, *p* < 0.01, *d* = 1.38, with decisive evidence in support of the alternative, BF_10_ = 218.91.

#### Signal detection theory analyses

Signal detection theory was used to calculate how *d’* (sensitivity) and *c* (criterion) changed across conditions.^2^

#### Sensitivity (d’)

A one-way ANOVA showed no significant difference in *d’* across conditions, *F*(2, 57) = 0.94, *p* < 0.40, *η*^2^_*p*_ = 0.03. As there was no significant difference in the overall ANOVA the data were not analysed further.

#### Criterion (c)

A one-way ANOVA showed a significant difference in criteria across conditions, *F*(2, 57) = 17.96, *p* < 0.01, *η*^2^_*p*_ = 0.39. Planned t-tests showed that participants were more willing to say that a target was present in the Bad condition in comparison with the Control condition, *t*(38) = 6.23, *p* < 0.01, *d* = 1.97, with decisive evidence in support of the alternative, BF_10_ = 40,541.70. The results did not show a difference in *c* between the Control and the Good condition, *t*(38) = 0.05, *p* = 0.96, *d* = 0.02, with substantial evidence in support of the null, BF_10_ = 1/3.24. Participants were more willing to say that a target was present in the Bad condition in comparison with the Good condition, *t*(38) = 4.92, *p* < 0.01, *d* = 1.56, with decisive evidence in support of the alternative, BF_10_ = 1008.12.

#### RTs

Mean correct RTs were entered into a 2 × 3 ANOVA with factors of Target Presence (Present vs Absent) and Condition (Bad, Control and Good). The results showed there to be a main effect of Target Presence, *F*(1, 57) = 76.80, *p* < 0.001, *η*^2^_*p* =_ 0.57. Participants were slower to respond when the target was absent compared to when it was present. The results did not show a difference of Condition, *F*(2, 57) = 1.69, *p* = 0.19, *η*^2^_*p* =_ 0.06. However, there was a significant interaction between Target Presence and Condition, *F*(2, 57) = 7.36, *p* = 0.001, *η*^2^_*p*_ = 0.21. When the target was absent, participants took longer to respond in the Bad condition compared to the other conditions.

#### Participant self-reported motivation

To examine participants self-reported motivation, data from the three Likert Questions were analysed using three one-way ANOVAs. There was a significant difference in responses for Question 1, *F*(2, 57) = 7.81, *p* < 0.01, *η*^2^_*p*_ = 0.22, Question 2, *F*(2, 57) = 5.13, *p* < 0.01, *η*^2^_*p*_ = 0.15, and Question 3, *F*(2, 57) = 4.57, *p* = 0.01, *η*^2^_*p*_ = 0.14. In all questions, participants reported higher scores in the Good condition, followed by the Control and then the Bad condition (see Table 1).

**Table 1:** Average Likert ratings across all conditions

	Bad	Control	Good
Question 1	3.8 (0.6)	4.1 (0.6)	4.5 (0.6)
Question 2	3.8 (0.6)	4.1 (0.8)	4.4 (0.6)
Question 3	4.1 (0.9)	4.3 (0.9)	4.8 (0.4)

Standard deviation is in the parentheses

The results also examined whether the response criteria, *c*, for the Bad, Control and Good conditions correlated with their respective self-report Likert scales. As these were post hoc analyses, a Bonferroni correction was applied, giving an adjusted p-value of 0.006 (initial alpha = 0.05; nine comparisons). However, none of the correlations showed significant differences (see Table 2).

**Table 2:** Results from the correlations between Response Criteria and the three Likert questions in each condition

Correlation	Pearson’s *r*	*P*-value	BF_10_
Bad—Question 1	0.003	0.99	1/3.61
Bad—Question 2	0.10	0.69	1/3.36
Bad—Question 3	− 0.13	0.60	1/3.16
Control—Question1	0.05	0.85	1/3.55
Control—Question2	0.46	0.04	1.91
Control—Question3	0.31	0.19	1/1.60
Good—Question 1	0.10	0.67	1/3.32
Good—Question 2	0.07	0.77	1/3.47
Good—Question 3	− 0.43	0.06	1.46

### Discussion

Experiment 1 manipulated the type of false feedback people were presented. Unsurprisingly, data from the self-reported Likert questions showed that people reported feeling more motivated in the Good condition followed by the Control and the Bad condition. Importantly, the feedback also had an effect on how participants searched for a cancer. Participants made fewer miss errors in the Bad compared to the Good and Control condition. However, participants also showed a greater proportion of false alarms in the Bad condition compared to the other conditions. The SDT data showed that there was little effect of false feedback on sensitivity. Instead, the false feedback led to a shift in participants response criteria to adopt a more liberal response bias in the Bad condition. This change in response criteria can explain the reduction in miss errors and increase in false alarms as people were more willing to respond ‘target present’ with bad feedback to try and reduce their reported miss error rates. The response time data also showed that participants took longer to respond before declaring a target was absent in the Bad condition. That is, participants were less likely to quit the task before thoroughly searching the display. This careful search may also have contributed to the reduction of miss errors in the Bad Condition.

Obviously, reducing miss errors in an applied task such as mammography is important to achieve. However, the corresponding increase in false alarms is not ideal, given that it would unnecessarily increase the number of women recalled for further examination. Instead, a better situation would be a reduction in miss errors, without an increase in false alarms. Given that the feedback in this experiment solely centred on miss errors, we examined whether giving participants feedback about both their *miss errors and false alarms* would retain the reduction in miss errors without increasing the number of false alarms. This was investigated in Experiment 2.

## Experiment 2

### Method

#### Participants

Forty participants took part in the experiment, with twenty participants per condition. Participants did not take part in any of the other experiments.

#### Stimuli and procedure

The Experiment was identical to that of Experiment 1, except for the following. As there was little difference between the Control condition and Good condition in Experiment 1, we omitted the Good condition in Experiment 2. Participants took part in one of two conditions: a Control condition and a ‘Bad’ feedback condition where they were told their performance was worse than average in terms of both miss errors and false alarms. For miss errors participants were given the same false feedback as in Experiment 1. In this condition, participants were also given false feedback about false alarms. Here participants were presented the message “Most people’s error rates for absent trials are 5%. Yours are *y*%”, where *y* was equal to 18%, 16%, 17%, and 15% respectively after each block. Participants were presented with the miss error and false alarm feedback at the same time at the end of each block. Participants were not asked to complete the Likert questions in this experiment.

### Results

**Fig. 3:**
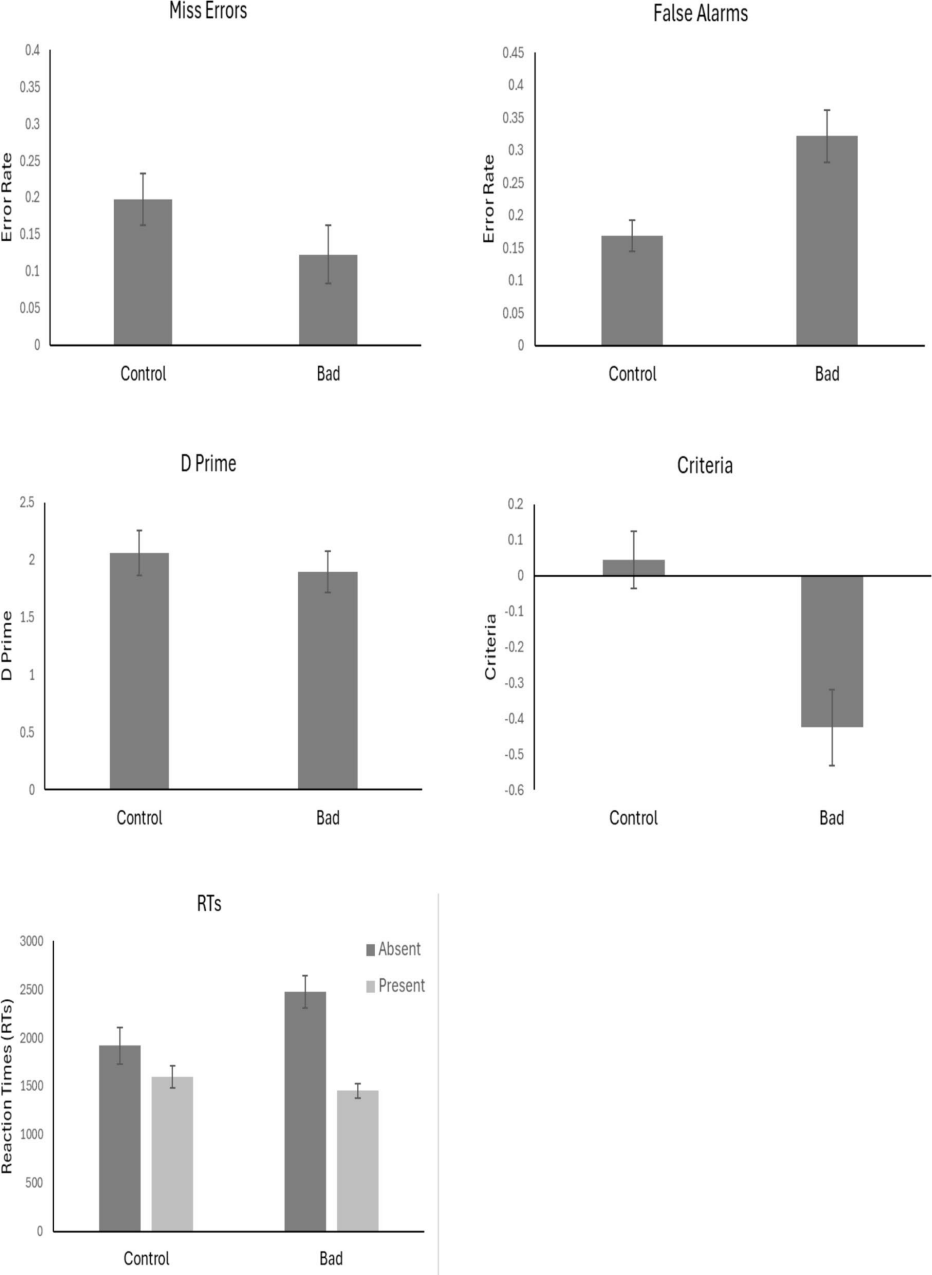
Mean values across conditions. *Note.* Error bars represent the standard error

The outlier procedure removed 1.6% of all data. Mean error rates, *d’, c* values and RTs for all conditions are presented in Fig. 3.

#### Miss errors

An independent t-test did not show a difference between miss errors in the Bad compared to the Control condition *t*(38) = 1.44, *p* = 0.16, *d* = 0.45, with anecdotal evidence in support of the null, BF_10_ = 1/1.44.

#### False alarms

An independent t-test showed that there was a greater proportion of False Alarms in the Bad condition compared to the Control condition, *t*(38) = 3.27, *p* < 0.01, *d* = 1.03, with strong evidence in support of the alternative, BF_10_ = 15.70.

#### Sensitivity (d’)

An independent t-test did not show a difference in *d’* between the Bad and Control conditions, *t*(38) = 0.61, *p* = 0.54, *d* = 0.19, with anecdotal evidence in support of the null, BF_10_ = 1/2.79.

#### Criterion (c)

An independent t-test showed that criteria was higher in the Control condition compared to the Bad condition, *t*(38) = 3.52, *p* < 0.01, *d* = 1.11, with strong evidence in support of the alternative, BF_10_ = 27.9.

#### RTs

Mean correct RTs were entered into a 2 × 2 ANOVA with factors of Target Presence (Present vs Absent) and Condition (Bad vs Control). The results showed there to be a main effect of Target Presence, *F*(1, 38) = 31.78, *p* < 0.001, *η*^2^_*p*_ = 0.46. Participants were slower overall to respond when the target was absent compared to when it was present. The results did not show a difference of Condition, *F*(1, 38) = 1.67, *p* = 0.21, *η*^2^_*p*_ = 0.04. However, the Target Presence x Condition interaction was significant, *F*(1, 38) = 8.76, *p* = 0.005, *η*^2^_*p*_ = 0.19. There was a greater slowing of RTs when targets were absent compared to present in the Bad condition compared to the Control condition.

### Discussion

Experiment 1 found that giving participants feedback in relation to their miss errors reduced miss errors in the Bad condition but also increased false alarms. Experiment 2 examined whether feedback given to participants about both their miss errors and false alarms reduced both types of error rates. However, the results showed little benefit. False alarms in the Bad condition were higher than in the Control condition, with no reduction in miss errors. The data suggested that the Bad feedback led to a change in response criteria, where participants adopted a more liberal response criterion and a slowing of RTs on target-absent trials in the Bad condition, similar to Experiment 1. However, unlike Experiment 1 this change in response bias did not lead to any benefit of cancer detection rate.

Experiments 1 and 2 found that giving people false feedback in an LP search task influenced their performance. Experiments 3 to 5 investigated effects of other motivational factors on LP search. Experiments 3 and 4 examined whether signing a pledge influenced LP search by priming participants to perform better in the search task. Experiment 3 examined this using a letter visual search that was lacking inherent motivation, while Experiment 4 used a mammogram search task that had more meaningful implications.

## Experiment 3

### Method

#### Participants

Forty participants took part in the experiment, with twenty participants per condition. Participants did not take part in any of the other experiments.

#### Stimuli and procedure

**Fig. 4:**
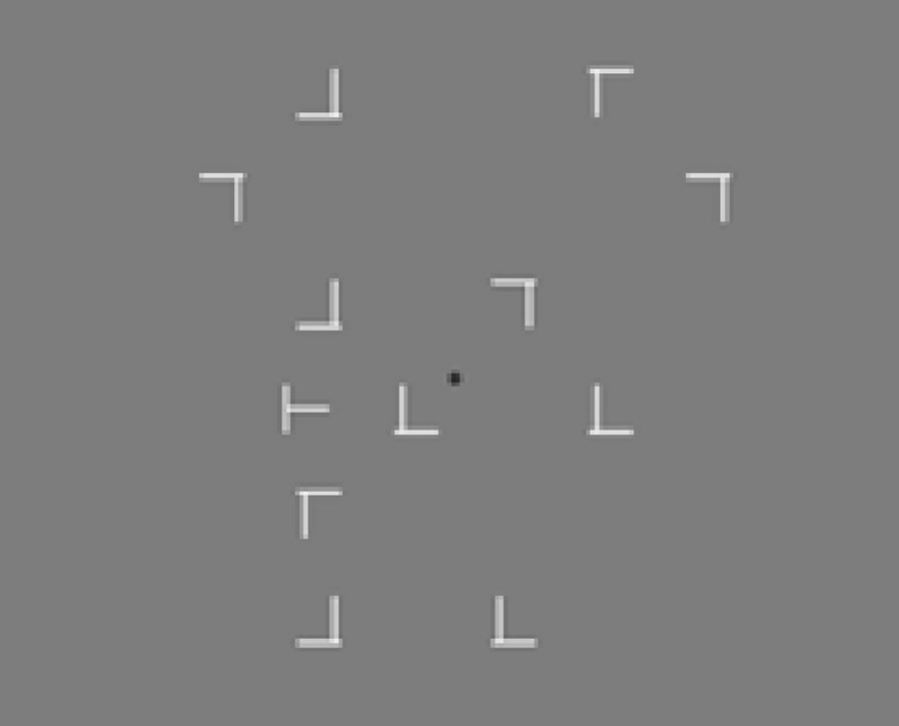
Example of stimuli used in Experiment 3

The experiment was the same as Experiment 1, except that here participants were asked to search through a letter visual search task (see Fig. 4 for examples). The stimuli were white coloured rotated Ts and Ls, in which the vertical lines of the Ls were slightly offset from its horizontal line (see Fig. 1). All stimuli were presented on a grey background. On each trial, there were always 12 stimuli presented (on ‘target-absent’ trials—12 distractors; on ‘target present’ trials—1 target and 11 distractors).

Participants were asked to take part in one of two conditions: a Sign and a No-Sign condition. In each condition there were 500 trials. Twenty of these trials contained the target, T. The other 480 trials were target-absent trials and did not contain a target. This resulted in a target prevalence rate of 4%. Participants were informed that the target would appear rarely. Stimuli were presented randomly within an invisible 6 × 6 matrix. To begin each trial, a blank screen appeared for 500 ms and was followed by a central fixation point for 500 ms. In each trial participants were shown a blank screen for 500 ms followed by a fixation dot for 1 s. They were then shown the search display, containing the letter stimuli presented in an invisible 6 × 6 matrix. Participants were asked to respond to whether a target letter, T, was present or absent by pressing the letter ‘m’ if the target was present and the letter ‘z’ if the target was absent. The L stimuli were presented randomly in one of four orientations (0 degrees, 90 degrees, 180 degrees or 270 degrees with equal probability). The T stimuli was on its side and could appear with the bottom of the T facing to the left or facing to the right. Stimuli were visible until participants made a response, at which point the next trial started. Participants were not given any feedback as to whether they had made the correct response.

In the Sign Condition, participants were asked to read and sign a ‘Pledge of Honour’ statement prior to beginning the experiment. The statement read:

*This research investigates how people search for a rare target. Searching for a rare target has important consequences for real life search tasks such as searching for a cancer in a mammogram or weapon in x-rayed baggage at the airport. Therefore, it is very important to find the target.*

They were then given the pledge that read: “*I declare that I will search the display to the best of my ability and respond as accurately as I can as to whether the target is there or not*” and asked to sign if they agreed. All participants in the Sign condition signed the pledge. In the No-Sign condition, participants were not shown the pledge statement and continued straight to the experiment without signing anything.

### Results

**Fig. 5:**
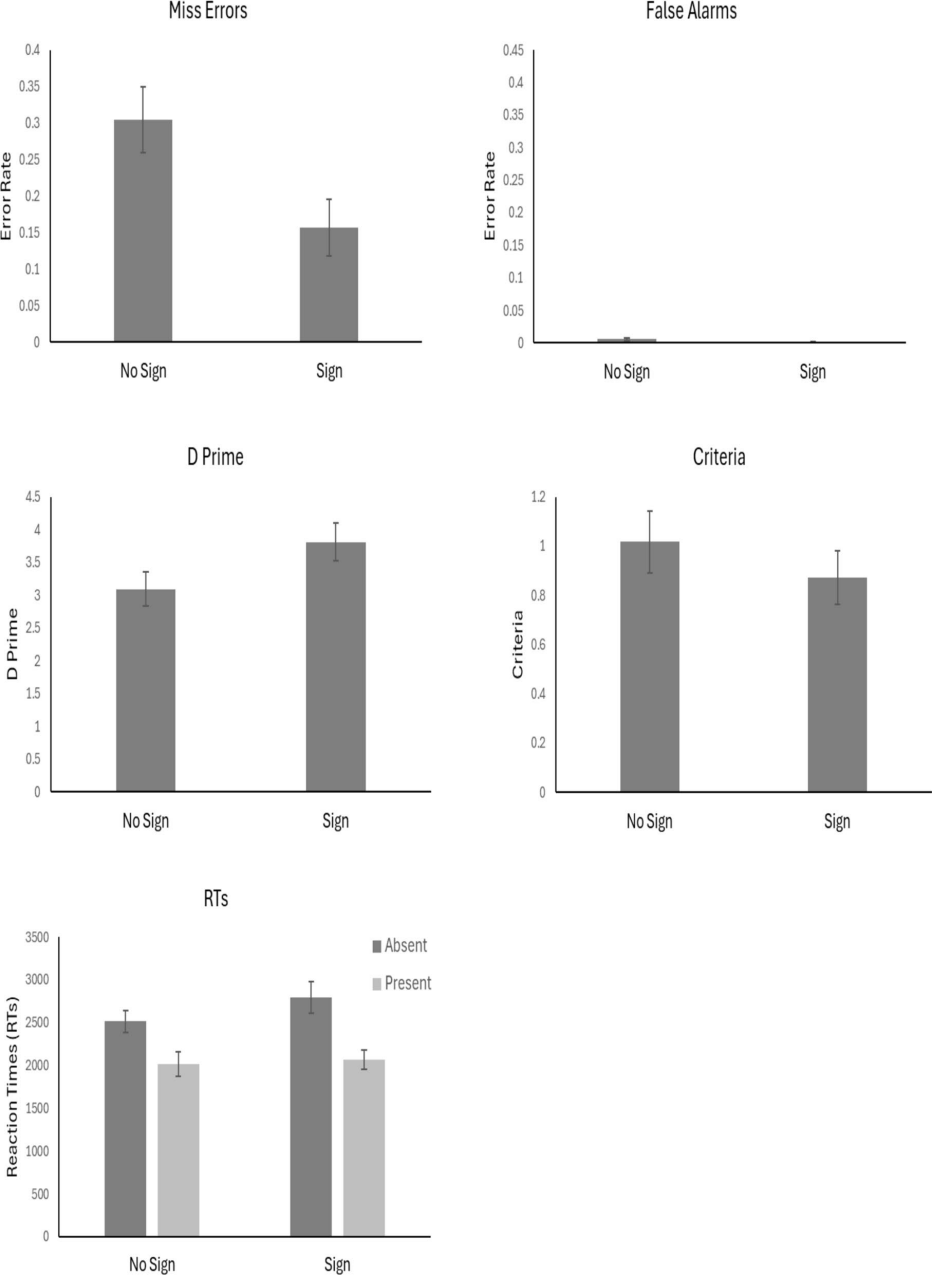
Mean values across conditions. *Note.* Error bars represent the standard error

The outlier procedure removed 0.78% of all data. Mean error rates, *d’*, *c* values and RTs for all conditions are presented in Fig. 5.

#### Miss errors

An independent t-test showed that participants made fewer miss errors in the Sign condition in comparison to the No-Sign condition, *t*(38) = 2.49, *p* = 0.017, *d* = 0.79, with substantial evidence in support of the alternative BF_10_ = 3.28.

#### False alarms

An independent t-test did not show a difference in False alarms between the Sign condition and the No-Sign condition, *t*(38) = 1.65, *p* = 0.11, *d* = 0.52, with anecdotal evidence in support of the null, BF_10_ = 1/1.12.

#### Sensitivity (d’)

An independent t-test showed that d’ was greater in the Sign condition in comparison to the No-Sign condition, *t*(38) = 3.31, *p* < 0.01, *d* = 1.05, with strong evidence in support of the alternative, BF_10_ = 17.38.

#### Criterion (c)

An independent t-test did not show a difference in *c* between the Sign condition and the No-Sign condition, *t*(38) = 1.65, *p* = 0.11, *d* = 0.52, with anecdotal evidence in support of the null, BF_10_ = 1/1.11.

#### RTs

Mean correct RTs were entered into a 2 × 2 ANOVA with factors of Target Presence (Present vs Absent) and Condition (Sign vs No Sign). The results showed there to be a main effect of Target Presence, *F*(1, 38) = 32.17, *p* < 0.001, *η*^2^_*p*_ = 0.46, in which participants were slower to respond when the target was absent compared to when it was present. The results did not show a difference of Condition, *F*(1, 38) = 0.93, *p* = 0.34, *η*^2^_*p*_ = 0.02. Neither did the results show a significant interaction between Target Presence and Condition interaction, *F*(1, 38) = 1.16, *p* = 0.29, *η*^2^_*p*_ = 0.03.

### Discussion

Experiment 3 showed that there was a benefit of having participants sign a pledge in LP search. Participants missed fewer targets in the Sign condition compared to the No-Sign condition. In accordance with the previous research, false alarm rates for these stimuli were minimal, given that the target was perceptually easy to identify (e.g. Kunar & Humphreys, 2006; Kunar et al., 2017). Surprisingly, the results did not show a difference in criteria across conditions and instead the benefit in miss errors occurred because people showed greater sensitivity in the Sign condition. It may be that signing the pledge led to an increase in the quitting threshold leading to more careful consideration before terminating search, in accordance with the MDM account of LP (Wolfe & Van Wert, 2010). There was some numerical evidence of this as RTs in the Sign condition were slower than in the No-Sign condition for target-absent trials. However, we hold this difference lightly as it was not significant.

The results of Experiment 3 showed that signing a pledge in a letter visual search task led to a reduction of the LP effect. Encouraging people to engage in the task provided greater motivation, and better search in a task, that was otherwise devoid of intrinsic motivation. Experiment 4 examined whether a similar benefit occurred when participants were presented with a mammogram search task. Previous studies have shown that presenting people with medical images can affect their motivation and underlying cognitive mechanisms (e.g. Hollands & Marteau, 2013). If medical images already enhance motivational mechanisms, then participants may naturally be more motivated to do the task well in the both the Sign and No-Sign condition, rendering the motivation of signing a pledge negligible.

## Experiment 4

### Method

#### Participants

Forty participants took part in the experiment, with twenty participants per condition. Participants did not take part in any of the other experiments.

#### Stimuli and procedure

The experiment was the same as Experiment 3, except that here participants were asked to search for a cancer in a mammogram using the same stimuli reported in Experiments 1 and 2.

Participants took part in either the Sign or No Sign condition. In both conditions, participants undertook the training session and practice trials, before proceeding to the main experiment. In the Sign Condition, prior to the experiment participants were asked to read and sign the same ‘Pledge of Honour’ outlined in Experiment 3. All participants in the Sign condition signed the pledge. In the No-Sign condition, participants were not shown the pledge statement and continued straight to the experiment without signing anything.

### Results

**Fig. 6:**
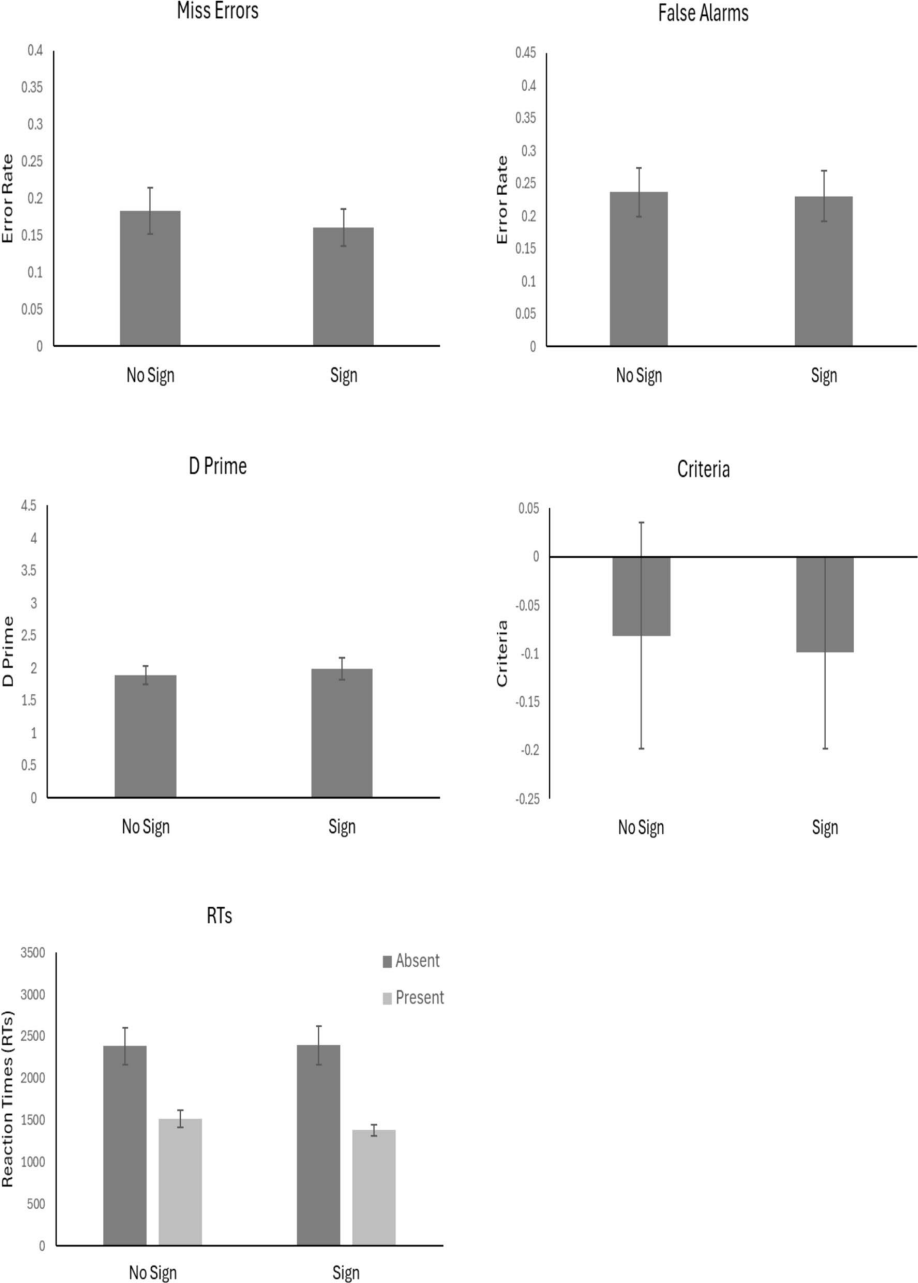
Mean values across conditions. *Note.* Error bars represent the standard error

The outlier procedure removed 0.5% of all data. Mean error rates, *d’, c* values and RTs for all conditions are presented in Fig. 6.

#### Miss errors

An independent t-test did not show a difference in Miss Errors between the Sign condition and the No-Sign condition, *t*(38) = 0.56, *p* = 0.58, *d* = 0.18, with anecdotal evidence in support of the null, BF_10_ = 1/2.86.

#### False alarms

An independent t-test did not show a difference in False alarms between the Sign condition and the No-Sign condition, *t*(38) = 0.12, *p* = 0.91, *d* = 0.04, with substantial evidence in support of the null, BF_10_ = 1/3.22.

#### Sensitivity (d’)

An independent t-test did not show a difference in *d’* between the Sign condition and the No-Sign condition, *t*(38) = 0.42, *p* = 0.68, *d* = 0.13, with substantial evidence in support of the null, BF_10_ = 1/3.02.

#### Criterion (c)

An independent t-test did not show a difference in *c* between the Sign condition and the No-Sign condition, *t*(38) = 0.12, *p* = 0.91, *d* = 0.04, with substantial evidence in support of the null, BF_10_ = 1/3.22.

#### RTs

Mean correct RTs were entered into a 2 × 2 ANOVA with factors of Target Presence (Present vs Absent) and Condition (Sign vs No Sign). The results showed there to be a main effect of Target Presence, *F*(1, 38) = 47.54, *p* < 0.001, *η*^2^_*p*_ = 0.56, in which participants were slower to respond when the target was absent compared to when it was present. The results did not show a difference of Condition, *F*(1, 38) = 0.10, *p* = 0.75, *η*^2^_*p*_ = 0.003. Neither did the results show a significant interaction between Target Presence and Condition interaction, *F*(1, 38) = 0.29, *p* = 0.59, *η*^2^_*p*_ = 0.008.

### Discussion

Experiment 4 examined the effect of participants signing a pledge in a low prevalence cancer detection task. The data showed several important points. First, false alarms in this experiment were much greater than those observed in Experiment 3. This was expected given that the perceptual discrimination of the target was more complex than in a standard letter visual search task (e.g. Kunar et al., 2017, see also Experiments 1 and 2). Second, in contrast to Experiment 3, there was no benefit of having participants sign a pledge. Signing a pledge led to no reduction in miss errors across conditions, nor in false alarm rates. Neither were there any differences to sensitivity, criteria or RTs.

Taken together, Experiments 3 and 4 showed that having participants sign a pledge affected performance in a meaningless visual search task but not in a more meaningful mammogram search. There are several differences between these two search tasks which could have influenced the results. For example, mammogram images are perceptually more complex to search with a different decision component, given the task difficulty, compared to letter visual search (as reflected in the higher false alarm rates). Furthermore, given the medical nature of the task, it could be that participants were inherently motivated to do the task well in both the Sign and No-Sign conditions. This may have eclipsed any motivational benefit of the pledge.

We investigated this latter point in Experiment 5, where we further assessed whether the medical motivation of the task affected the LP effect. Here, participants were either explicitly told the healthcare importance associated with the task (in the Motivated condition) or given neutral information. It was investigated whether this extra, inherent motivation led to a reduction in miss errors, or whether the motivation provided by the medical stimuli, was already at ceiling.

## Experiment 5

### Method

#### Participants

Forty participants took part in the experiment, with twenty participants per condition. Participants did not take part in any of the other experiments.

#### Stimuli and procedure

The stimuli and procedure were the same as those of Experiment 4, except for the following changes. Participants took part in one of two conditions: a Neutral condition and a Motivated condition. In both conditions, participants undertook the training session and practice trials. Participants were then given a passage to read before they completed the experimental stage (see Table 3). In the Neutral condition, participants read a passage, detailing objective facts about the procedures involved in searching for breast cancers in mammograms. In the Motivated condition, participants read a passage that contained similar content, but with more emotive language and information regarding the importance of research such as this, and the dangers of misdiagnosis in mammography. All participants then completed the mammogram visual search task. For all participants, there were four block breaks that automatically occurred following every 100 trials. At this point, participants were given the message “Take a break—when you are ready press enter to continue”. Participants could take as long as they wanted to break before continuing onto the next block.

**Table 3:** Message presented to participants at the start of each condition

Neutral	Motivated
Research shows that breast screening reduces breast cancer-related deaths by around 1300 individuals a year in the UK. This screening usually utilizes mammograms as a method to detect breast cancer. A mammogram can either be used to detect tumours and masses that cannot be felt, or to check for breast cancer after a lump or another symptom has already been reported. The procedure involves taking x-ray images of breast tissue and visually searching for a cancerous mass. As this involves visually searching the mammograms, there is a risk of errors in diagnoses, that research shows to be higher with rarer targets such as breast cancer tumours	Generally, the survival rate for breast cancer is very good, and is increasing each year with advances in the medical community. Research shows that breast screening reduces breast cancer-related deaths by around 1300 individuals a year in the UK. This screening usually utilizes mammograms as a method to detect breast cancer, taking x-ray images of breast tissue and visually searching for a cancerous mass. Nearly 8 out of every 1000 mammograms will screen positive for breast cancer, meaning that research into factors surrounding visual search with rare targets is crucial. With research such as this, we hope to eventually increase the efficiency and accuracy of mammogram-reading and therefore reduce the prevalence of missed or wrong diagnoses. Incorrect diagnoses can often lead to unnecessary invasive procedures and cause patient anxiety and emotional stress, and in the worst of cases, decrease the likelihood of the breast cancer being detected before it is too late. It is therefore vital that you search these images to the best of your ability, as research such as this may contribute to the improvement of mammogram-reading and subsequently may help to save lives!

### Results

**Fig. 7:**
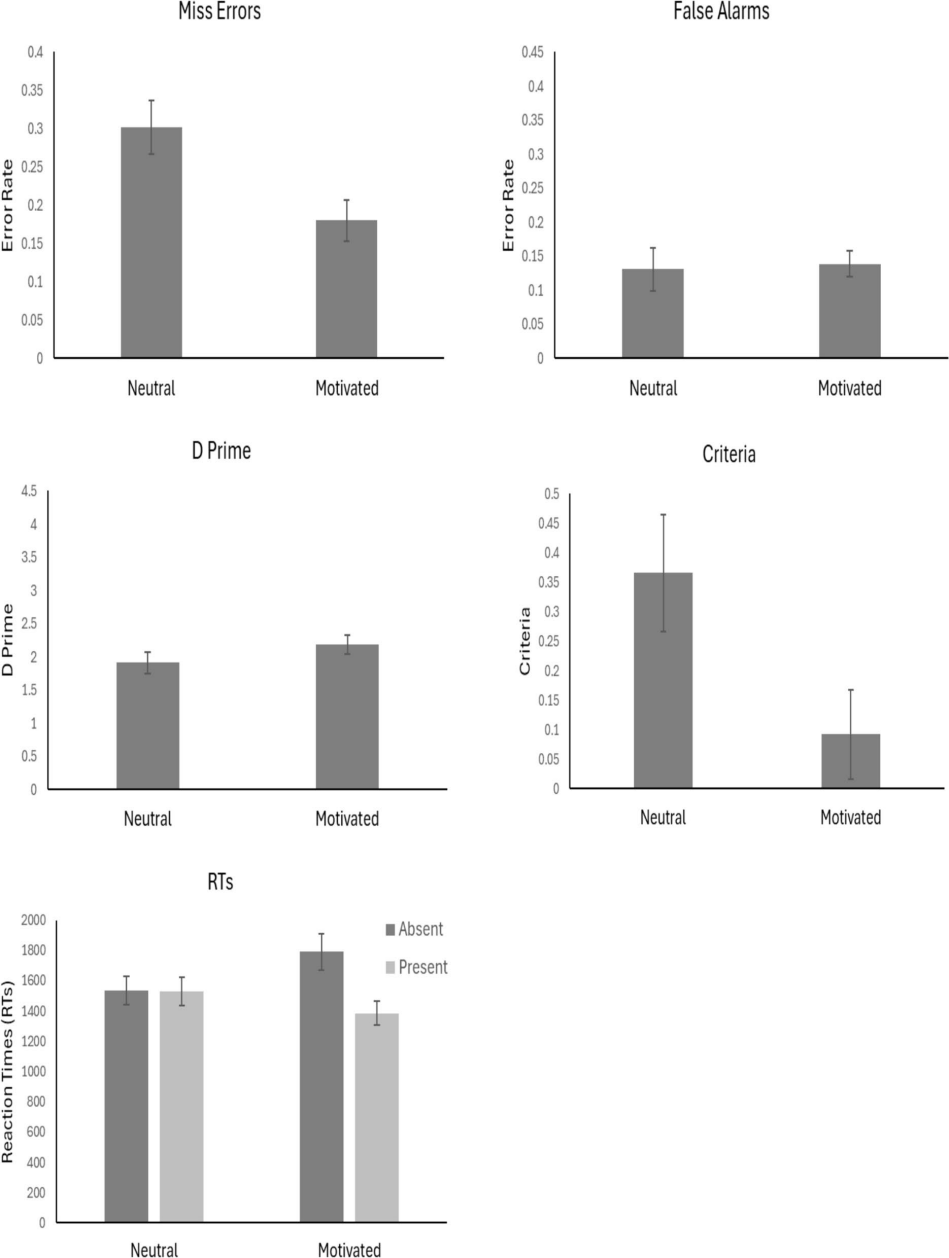
Mean values across conditions. *Note.* Error bars represent the standard error

The outlier procedure removed 0.6% of all data. Mean error rates, *d’, c* values and RTs for all conditions are presented in Fig. 7.

#### Miss errors

An independent t-test showed that there were fewer miss errors in the Motivated condition in comparison to the Neutral condition *t*(38) = 2.73, *p* < 0.01, *d* = 0.87, with substantial evidence in support of the alternative, BF_10_ = 5.19.

#### False alarms

An independent t-test did not show a difference in False alarms between the Neutral and Motivated condition, *t*(38) = 0.22, *p* = 0.83, *d* = 0.07, with substantial evidence in support of the null, BF_10_ = 1/3.19.

#### Sensitivity (d’)

An independent t-test did not show a difference in *d’* between the Neutral and Motivated condition, *t*(38) = 1.28, *p* = 0.21, *d* = 0.41, with anecdotal evidence in support of the null, BF_10_ = 1/1.70.

#### Criterion (c)

An independent t-test showed that criteria was higher in the Neutral condition compared to the Motivated condition, *t*(38) = 2.19, *p* = 0.04, *d* = 0.69, with anecdotal evidence in support of the alternative, BF_10_ = 1.96.

#### RTs

Mean correct RTs were entered into a 2 × 2 ANOVA with factors of Target Presence (Present vs Absent) and Condition (Neutral vs Motivation). The results showed there to be a main effect of Target Presence, *F*(1, 38) = 6.55, *p* = 0.02, *η*^2^_*p*_ = 0.15, in which participants were slower overall to respond when the target was absent compared to when it was present. The results did not show a difference of Condition, *F*(1, 38) = 0.24, *p* = 0.63, *η*^2^_*p*_ = 0.006. However, the Target Presence x Condition interaction was significant, *F*(1, 38) = 6.23, *p* = 0.02, *η*^2^_*p*_ = 0.14. There was a greater slowing of RTs when targets were absent compared to present in the Motivated condition compared to the Neutral condition.

### Discussion

The results were clear. Although the results did not show a difference in false alarm rates, participants in the Motivated condition showed fewer miss errors in comparison to those in the Neutral condition. The SDT data revealed this was due to a shift in response criteria in the Motivated condition. Participants in this condition, were more willing to respond that a target was present in comparison to the Neutral condition. The results did not show a difference in sensitivity between conditions. The response time data also showed that participants were slower to respond on target-absent trials in the Motivated condition. That is, in this condition participants were less likely to quit their search before thoroughly searching the display, leading to a reduction of miss errors when the cancer was present. Previous work has found that manipulating external rewards could be affective in altering performance in a low prevalence search (e.g. Navalpakkam et al., 2009). These data suggest that low prevalence search can also be affected by manipulating intrinsic factors within motivation. We discuss this further in the General Discussion.

## General discussion

This paper investigated the effect of false feedback and motivation and on LP search. Experiments 1 and 2 examined the effects of false feedback. Interestingly, positive feedback, where participants were told they were doing well, did little to affect task performance (Experiment 1). However, miss errors were reduced, if participants were told they were making too many miss errors. Unfortunately, this was also accompanied by a rise in false alarms. Giving people false feedback about both miss errors and false alarms did little to benefit search (Experiment 2). In this case, we observed increased false alarm rates with no corresponding drop in miss errors. Our results replicate and extend the effects of Schwark et al. (2012), showing that the effects of false feedback occurred in the absence of external reward manipulations. However, with a reduction in miss errors, there was also an increase in false alarms. In an applied setting this would have the benefit of more cancers being detected, but with more women being unnecessarily recalled for further tests. This increase in recalled women is problematic, putting increased pressure on already burdened healthcare systems and causing heightened anxiety in women who have been affected, lasting for up to a year (Aro, 2000). It also has implications for future medical screening with affected women more likely to delay or even decline participation (Kahn & Luce, 2003).

Experiments 3–5 examined the effects of other internal motivational factors on LP search. Previous work found that external incentives, such as monetary rewards led to a reduction in the LP effect (Navalpakkum et al., 2009; Hadjipanayi et al., 2023). The current data add to this knowledge, showing intrinsic motivation influenced LP search in different ways. Experiment 3 found that signing a pledge led to a reduction in the LP effect in a ‘meaningless’ search task. Previous research has shown that making a pledge activates self-identity cues, encouraging observers to be more engaged in an activity (Kettle & Häubl, 2011). A similar effect emerged here where signing a pledge led to a reduction in miss errors in a letter visual search task. However, the same did not occur when observers were asked to search mammogram images for a cancer. There are several potential reasons to explain the differences found between Experiments 3 and 4. For example, search through the mammogram images is perceptually more difficult than search in the letter visual search task, resulting in a different decision component. This is supported by the higher False Alarm rates witnessed in the mammogram search compared to the letter visual search, where participants mistook a ‘non-target’ area for a target. It could be that these more complex perceptual and decision factors negated any benefits of the pledge with the medical stimuli. Another potential reason could be that medical images inherently prime motivation (e.g. Hollands & Marteau, 2013). In this case, participants in both the pledge and non-pledge mammogram tasks, were equally motivated to do the task, rendering the benefit from the pledge ineffective. Experiment 5 further examined whether motivation was at ceiling in the mammogram search task or whether emphasising the medical importance, reduced the LP effect. The results showed that highlighting the importance of the task led to fewer missed cancers, with a shift to a more liberal response bias.

Taken together, the data showed that feedback and intrinsic motivation had an impact on LP search. According to the MDM theory of LP search, the LP effect occurred due to (i) a lowering of the quitting threshold for terminating search and (ii) a change in response criteria to a more conservative bias (Wolfe & Van Wert, 2010). Our results showed that increased motivation produced a liberal response criteria shift, meaning that participants were more willing to respond a target was present.^3^ In our studies, the liberal response bias countered the conservative shift in response criteria driven by low prevalence, leading to a reduction in the LP Effect. It would be up to future work to assess whether increasing motivation in a clinical setting would provide the same benefit. It may be that professionals in a healthcare setting are already motivated by the importance of their work. However, worryingly, recent research has found that healthcare professionals reported low levels of work motivation (Karaferis et al., 2022) and that low motivation may be considered the second greatest workplace challenge in healthcare settings (the first being staff shortages, Mathauer & Imhoff, 2006). Given the implications from our work, it becomes vital to examine ways to improve motivation of healthcare employees in a bid to achieve optimal performance in LP tasks like mammography.

Of course, there are other important differences between the work presented here and those in a clinical setting. For example, the prevalence rate used in our experiments was higher than the prevalence rate of cancer in clinical mammography. Furthermore, our participants, by design, were not medical experts and missing a cancer in these experiments would not have the same consequences as those in a clinical setting. It could be that these natural consequences provide increased motivation in an applied setting, above that which we could create in the lab. Future work is needed to investigate this. However, for now, our study shows that intrinsic motivation is a fruitful avenue of research for LP search.

Regarding the effect of false feedback, please note that our studies did not examine participants’ meta-awareness of their search performance and how it matched the feedback they were told. As the false feedback error rates were chosen to be plausible^4^ it could be that participants were more accepting of the false feedback compared to if they were told their performance was much worse than reality (for example, if the false feedback gave error rates that was double or triple the amount of the actual errors participants made). In this case it is not known if people would still accept the feedback and change their behaviour accordingly. Further studies are needed to investigate this.

Of final note, procedures like mammography are benefitting from recent developments in Artificial Intelligence (AI). CAD (Computer Aided Detection) use algorithms to highlight suspicious items to healthcare professionals and using AI-CAD as a supporting reader has been shown to improve the workflow in a mammography screening task (Ng et al., 2023). However, although CAD reduces the LP effect when accurate, it leads to an over-reliance effect, with increased missed cancer rates when inaccurate (Kunar et al., 2017). Furthermore, what people are told about CAD, and changes to the way it’s presented, affect peoples’ over-reliance (Kunar & Watson, 2023; Kunar, 2022; Patterson & Kunar, 2024; Kunar et al., 2024). The addition of AI may also affect motivation. For example, introducing AI could lead to cognitive loafing where human readers, rely more on AI recommendations, feeling less incentivised to do the task. It is therefore crucial to examine how AI recommendations affect motivation and the consequences this has for clinical tasks.

## Data Availability

The datasets generated and analysed during the current study are available in the Open Science Framework repository, https://osf.io/2qwka/.

## Abbreviations

LP: Low Prevalence
HP: High Prevalence
CAD: Computer-Aided Detection
ANOVA: Analysis of Variance
RTs: Reaction Times
AI: Artificial Intelligence
DDSM: Digital Database for Screening Mammography

## Footnotes

1 In the majority of our experiments we used a lab-based mammogram search task that has been specially designed for use with participants who have no medical training (e.g. Drew et al., 2020; Kunar, 2022; Kunar et al., 2017; Kunar et al., 2021; Kunar et al., 2024; Patterson & Kunar, 2024). This has the advantage of allowing recruitment of sufficient participants to achieve experimental power (which is difficult to do if recruiting radiologists with busy workloads). Given that medical and non-medically trained observers have the same underlying search process, (Wolfe et al., 2016) this method is well established in the field.

2 False alarm or miss error rates of 0 and 1 were adjusted using the formulas 1/2*n* and 1-(1/2*n*), where *n* = the number of trials (Macmillan & Kaplan, 1985, see also Russell & Kunar, 2012, Wolfe et al., 2007, and Kunar et al., 2021; 2024; Patterson & Kunar, 2024, who used this procedure).

3 This occurred in all experiments apart from in Experiment 3, where there was no difference in criteria.

4 Miss rates have been known to reach 30–40% in LP search (e.g. Kunar et al., 2010; Wolfe et al., 2005).

## Declarations

### Open Practices Statement

The data for all experiments are available on the Open Science Framework (https://osf.io/2qwka/). The study design, hypotheses, and analytic plan were not pre-registered.
